# *PMM2* -CDG T237M Mutation in a Patient with Cerebral Palsy-Like Phenotypes Reported from South India

**DOI:** 10.1055/s-0043-1769494

**Published:** 2023-06-01

**Authors:** N. Sreedevi, N. Swapna, Santosh Maruthy, H.S. Meghavathi, Charles Sylvester

**Affiliations:** 1 Department of Speech-Language Sciences, All India Institute of Speech and Hearing, Mysore, Karnataka, India; 2 Department of Speech-Language Pathology, All India Institute of Speech and Hearing, Mysore, Karnataka, India; 3 Unit for Human Genetics, All India Institute of Speech and Hearing, Mysore, Karnataka, India

**Keywords:** *PMM2* -CDG, cerebral palsy, mutation, South India

## Abstract

Congenital disorder of glycosylation (CDG) is an autosomal recessively inherited disorder. Hypotonia, stroke-like episodes, and peripheral neuropathy are also associated with the condition that typically develops during infancy. The patient, a 12-year-old girl born to healthy consanguineous parents, was diagnosed with cerebral palsy as a child. The affected patient has hypotonia, inadequate speech, strabismus, and developmental delay with mild mental retardation, which are key symptoms of CDG. Whole-exome sequencing (WES) identified the known missense pathogenic variant *PMM2* c.710 C > T, p.T237M in the patient coding for the phosphomannomutase 2 (PMM2) confirming molecular testing of CDG. The patient's parents carried heterozygous *PMM2* c.710 C > T variants. This study highlights the importance of WES in patients with a developmental disability or other neurological conditions, which is also useful in screening risk factors in couples with infertility or miscarriage issues.

## Introduction

Congenital disorder of glycosylation (CDG) type Ia (CDG-Ia) [MIM #212065] is an autosomal recessive disorder of protein *N* -glycosylation characterized by a phosphomannomutase (PMM) deficiency and mutations in the ( *PMM2)* gene [MIM #601785] located on chromosome 16p13 that causes CDG ( *PMM2* -CDG) type Ia. 1 2 *PMM2* -CDG is characterized by central nervous system dysfunction and multiorgan failure. 3 *PMM2* gene catalyzes the isomerization of mannose-6-phosphate to mannose-1-phosphate, which is a precursor to GDP-mannose necessary for the synthesis of dolichol-pyrophosphate-oligosaccharides in the early steps of *N* -glycan synthesis. 4 5 About 130 pathological mutations have been described in the *PMM2* gene according to the Human Gene Mutation Database (HGMD® professional 2021.3) affecting the oligosaccharide precursor transfer in the endoplasmic reticulum, and *PMM2* -CDG II affects the transfer in the Golgi apparatus. 6 7

Hypotonia, stroke-like episodes, and peripheral neuropathy are also associated with the condition that typically develops during infancy; young individuals with *PMM2* -CDG may have a moderate intellectual disability and unsteady or abnormal gait. 2 8 The childhood mortality rate is approximately 15 to 30%, and permanent neurological disabilities develop in surviving patients. 9 This case study describes the clinical and molecular findings of a young girl from South India affected with cerebral palsy (CP) phenotypes carrying *PMM2* -CDG mutation.

## Clinical Report

A 12-year-old female from South India was born preterm to healthy consanguineous parents. Her birth weight was 2,500 g and presented with a delayed birth cry. Since birth, she presented generalized hypotonia. No history of neonatal jaundice or neonatal hypoxia was reported. The average tone of the muscle motor sensor, an inaccurate gait pattern, and a bilateral flexed knee was observed. No ataxia or cerebellar syndrome was observed. Since an early age, she presented inadequate speech and language, learning difficulties with mild mental retardation and dependence on several activities of daily life, psychomotor and developmental delay, and inability to walk. No history of hearing difficulties was reported. The ophthalmological analysis showed squint vision. After explaining the purposes of the study, informed consent was obtained from the parents to perform molecular diagnosis, and to publish the data on the patient. Blood samples were collected from the patient and her family members by a Phlebotomist. Genomic DNA was extracted from peripheral blood by using PureLink Genomic DNA Mini Kit (Thermo Fisher Scientific, United States) according to the manufacturer's instructions. Whole-exome sequencing (WES) was performed for the proband. The exome libraries were constructed using the Ion Ampliseq exome RDY kit (Thermo Fisher Scientific, United States) and sequenced on the Ion Proton sequencing platform (Life Technologies, United States). Variants were called using the Torrent Variant Caller plug-in using the software console of the Torrent server. Variants were annotated using Ion Reporter (Thermo Fisher Scientific, United States) using the human reference genome (hg19). Pathogenicity of candidate variants was evaluated using Genome Aggregation Database ( https://gnomad.broadinstitute.org/ ), ClinVar ( https://www.ncbi.nlm.nih.gov/clinvar/ ), and literature review. Sanger sequencing was performed for variant validation of the proband and the parents by amplifying the *PMM2* region (rs80338708) using the primers (forward: 5′-ACAGATCTTCGCAAGAACATCGT-3′, reverse:5′- CACGTTAGGAGAACAGCAGTTCA-3′) for a total volume of 10 μL. An initial denaturation step at 95°C for 2 minutes was followed by 35 cycles of 98°C for 25 seconds, 67°C for 45 seconds for annealing, 72°C for 30 seconds for elongation, and final extension at 72°C for 7 minutes. The PCR products were evaluated using a 2% agarose gel electrophoresis. PCR products were labeled with BigDye Terminator v3.1 Cycle Sequencing Kit (Applied Biosystems, United States). The above-mentioned PCR primer ( *PMM2* Forward) was used as a sequencing primer and then analyzed by ABI 3500 Genetic Analyser (Applied Biosystems, United States). Sequence data were analyzed with Seqscape v3 software (Applied Biosystems, United States).

## Discussion

**Fig. 1:**
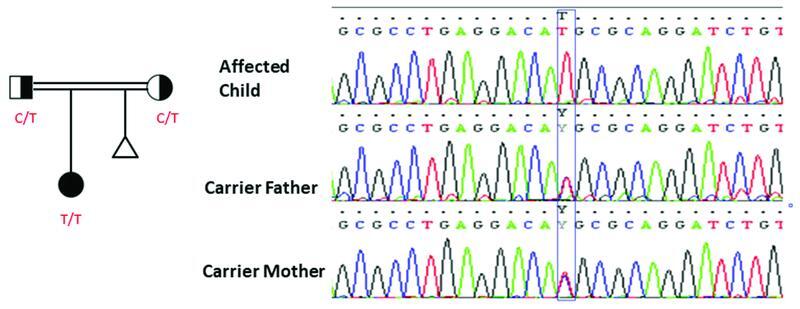
Pedigree and chromatograms generated by Sanger sequencing of the *PMM2* c.710C > T mutation detected in this study.

We describe the case of a girl with CP phenotypes who had missense variations in the *PMM2* gene that was identified by WES. The patient presented strong genotypic and phenotypic features of typical *PMM2* -CDG type 1a ( Table 1 ). WES identified the pathogenic homozygous variant [NM_000303.3] c.710C > T p.T237M (rs80338708) in the patient and also identified heterozygous variants in the parents ( Fig. 1 ). She exhibited age-inadequate speech and language skills; weakness in lower limbs was also observed in the patient. Spastic diplegia was observed that impairs the legs but does not usually affect the arms; but the patient in this study has weakness in the hands because of low muscle tone. Hip problems are also common in spastic diplegic patients. The spastic type of CP is more commonly associated with ocular abnormalities. 10 Strabismus was seen in the patient that is associated with neurodevelopmental disorders such as CP, Down's syndrome, intellectual disability, and white matter damage of prematurity. 11 12 13

**Table 1:** Clinical features and genotype

Sample name	CP_39A
Gender	Female
Age	12 years
Ethnic	South Asian
Mutation	*PMM2* c.710C > T237M (Homozygous)
Consanguinity	+
Preterm birth	+
Birth cry delay	+
Neonatal hypotonia	+
Neonatal hypoxia	–
Neonatal jaundice	–
Developmental delay	+
Mental retardation	+
Strabismus	+
Delayed speech and language	+
Hearing loss	–
Paralysis or weakness of limbs	+
Neuromuscular scoliosis	+
Renal involvement	Unknown
Liver involvement	Unknown
Heart problem	Unknown

On chromosome 16p13.2, mutations in *PMM2* gene that cause CDG-Ia were identified. 1 This work is the first to report the T237M variant of the *PMM2* gene in a patient with CDG-Ia phenotypes ( https://www.chop.edu/conditions-diseases/congenital-disorders-glycosylation-cdg ) in a South Indian girl ( Table 1 ). The parents carried the heterozygous recessive variant of *PMM2* c.710 C > T that was expressed as the homozygous genotype in their child (patient; Fig. 1 ). Mutations of the *PMM2* gene cause *PMM2* deficiency, which is the most common autosomal recessive CDG. 14 Several missense mutations associated with a recessive disease are observed in *PMM2* . 15 Predetermined maternal factors such as heredity, malnutrition, and metabolism may lead to physical or mental problems, or even death. 16 Premature birth and low birth weight, which are linked with numerous health problems later in life, also can be influenced by birth defects. However, factors such as adolescent pregnancy (<19 years), 17 advanced maternal age (>35 years), 18 and poor access to medical care contribute as well. 19 Also, birth defects are a contributing factor to some miscarriages and stillbirths. 20 For that reason, some factors affect the prenatal developmental stage of fetal abnormalities. In this study the mother has a history of one miscarriage after the patient was born ( Fig. 1 ). *PMM2* -CDG is not thoroughly diagnosed, since the phenotypes are similar to CP. Therefore, clinical awareness and molecular diagnosis should be performed to screen couples with infertility or miscarriage issues 21 and they should be appropriately counseled on the potential risk before subsequent pregnancies. In conclusion, we describe the first South Indian patient with autosomal recessive CDG-Ia with *PMM2* c.710 C > T, T237M mutation inherited from carrier parents in accordance with the *PMM2* -CDG type Ia. This study emphasizes the importance of considering WES in patients with developmental disabilities or other neurological conditions.
